# Pilot Project to Integrate Community and Clinical Level Systems to Address Health Disparities in the Prevention and Treatment of Obesity among Ethnic Minority Inner-City Middle School Students: Lessons Learned

**DOI:** 10.1155/2018/6983936

**Published:** 2018-03-26

**Authors:** Jessica Rieder, Agnieszka Cain, Erica Carson, Andrea Benya, Paul Meissner, Carmen R. Isasi, Judith Wylie-Rosett, Neal Hoffman, Colleen Kelly, Ellen J. Silver, Laurie J. Bauman

**Affiliations:** ^1^Department of Pediatrics, Children's Hospital at Montefiore, The Pediatric Hospital for Albert Einstein College of Medicine, Bronx, NY, USA; ^2^Department of Family and Social Medicine, Albert Einstein College of Medicine, Bronx, NY, USA; ^3^Department of Epidemiology and Population Health, Albert Einstein College of Medicine, Bronx, NY, USA

## Abstract

Effective obesity prevention and treatment interventions are lacking in the United States, especially for impoverished minority youths at risk for health disparities, and especially in accessible community-based settings. We describe the launch and pilot implementation evaluation of the first year of the *B'N Fit POWER* initiative as a middle school-based comprehensive wellness program that integrates weight management programming into existing onsite preventive and clinical services. Consistent with the existing implementation science literature, we focused on both the organizational structures that facilitate communication and the development of trust among stakeholders, students, and families and the development of realistic and timely goals to implement and integrate all aspects of the program. New implementation and programming strategies were developed and tested to increase the proportion of students screened, support the linkage of students to care, and streamline the integration of program clinical and afterschool components into routine services already offered at the school. We report on our initial implementation activities using the Standards for Reporting Implementation Studies (StaRI) framework using hybrid outcomes combining the Reach element from the RE-AIM framework with a newly conceptualized Wellness Cascade.

## 1. Introduction

While 17% of 2–19-year-old children and adolescents are affected by obesity in the United States [1], the most severe forms of obesity have increased, particularly among adolescents and non-Hispanic blacks [2]. Low SES are 1.35 times more likely to be obese and low SES ethnic minority adolescents are less likely to live in neighborhoods supportive of physical activity than higher SES Caucasian youths [3, 4]. Despite the fact that obesity in adolescence increases the risk for many chronic conditions, effective preventive and treatment interventions are lacking, especially for minority youths at risk for health disparities [5–7].

Intensive pediatric weight management programs featuring physical activity, nutrition, and sedentary behavior reduction counseling, parental involvement, and cultural tailoring [8–11] can decrease weight and risk for developing type 2 diabetes mellitus (DM) [6, 9, 12–15], yet most are offered in highly specialized treatment centers, are demanding of patients, and require highly trained staff [16]. Effective less-intensive, large-scale public health efforts that target youths in schools or communities tend to focus on prevention of obesity and while they engage and promote healthy lifestyle behaviors for large numbers of youths, they often lack comprehensive services and many have not demonstrated favorable effects on BMI [6, 17–21].

The Bronx Nutrition and Fitness Initiative for Teens (B'N Fit) was developed in 2005 by the Children's Hospital at Montefiore (CHAM) in partnership with the Mosholu Montefiore Community Center (MMCC) as a 9-month weight-loss program to address health disparities and treat obesity in adolescents from a low-income minority community. B'N Fit was a hybrid approach which included comprehensive obesity evaluation and treatment plans in a hospital ambulatory care service venue combined with community-based nutrition and physical activity education. Barriers to retention and achieving obesity-related recommendations included low motivation to change behaviors and the home and community environment. Nonetheless, B'N Fit decreased the rate of weight gain and changed behaviors in adolescents with high rates of severe obesity (67%) and obesity-related comorbidities (57%) [22]. However, youth feedback indicated that targeting adolescents with obesity and offering the program components at sites that were not in their normal routine affected attendance and retention rates. B'N Fit was perceived to be offered at inconvenient locations and times and attending could be stigmatizing.

Translating the program to a school setting for all students was a potential solution for addressing these issues. There is an abundance of literature evaluating school-based weight management intervention effectiveness, but there is relative paucity of implementation studies addressing how programs were implemented and whether their feasibility was evaluated and the Community Preventive Task Force has found insufficiency evidence to recommend school-based obesity programs to prevent or reduce overweight and obesity among children and adolescents [23–28]. Following the Standards for Reporting Implementation Studies (StaRI) reporting framework [29], this paper describes the approach used to launch and evaluate the implementation of the *B'N Fit POWER* initiative as a school-based comprehensive program that integrates weight management into programming accessible to all students with an emphasis on strategies for sustaining such programming in a low-resource middle school setting.

## 2. Methods

### 2.1. Description of the *B'N Fit POWER* Initiative

**Figure 1:**
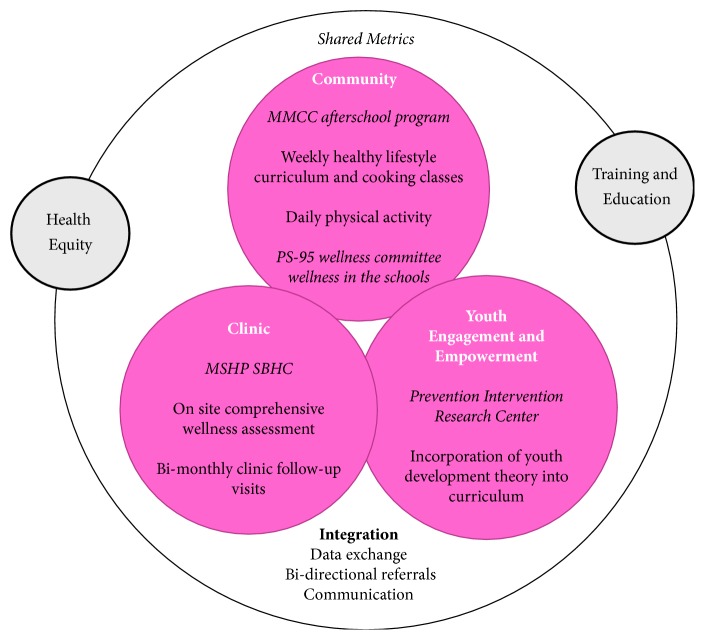
The *B'N Fit POWER* clinical and community integration framework. Adapted from the Clinical–Community Integration Framework by Dietz et al. [31].

The American health-care system is embracing the Triple Aim®—achieving better care for patients, better health for communities, and lower costs through health-care system improvement. It is developing new ways to integrate and coordinate services, emphasize patient engagement and patient-centered care, and develop new payment models that place value on population-based health outcomes rather than the volume of services [30]. The Clinical–Community Integration Framework as described by Dietz et al. [31] has been designed to address this needed change in the health-care system by integrating existing clinical and community services to provide improved coordination of services with an emphasis on close links between the patient and family unit as a model for preventing and treating obesity. *B'N Fit POWER* uses this framework and integrates several elements of successful intensive and school-based obesity prevention and treatment interventions by providing onsite medical care and youth-focused afterschool programming to teach and model healthy lifestyle behaviors and incorporating youth-centered outcome metrics to evaluate the program (Figure 1).

*B'N Fit POWER* is a voluntary multicomponent school-based wellness program that draws on Youth Development (YD) theory to emphasize adolescent strengths and promote positive adolescent development and health behaviors that lead to a healthy weight, rather than avoidance of risks and negative behaviors [32]. While studies have demonstrated that YD based curriculums, which foster a youth-centered, culturally appropriate, and psychologically safe atmosphere, can effectively decrease BMI and improve healthy lifestyle knowledge, behaviors, and attitudes; few concurrently evaluate health impact [33–38]. A school is an ideal setting to implement an integrated program with a YD emphasis because youths spend almost half of their waking hours in schools and may be more inclined to participate in activities that are supported by caring adults in their schools, especially if they have friends that also participate [19, 39]. *B'N Fit POWER's* implementation occurred at Public School/Middle School–95 (PS/MS-95), a kindergarten through 8th grade school that has both an onsite School-Based Health Center (SBHC) operated by the Montefiore School Health Program (MSHP) and an afterschool program run by the Mosholu Montefiore Community Center (MMCC). Working with existing services provided by involved stakeholders, the program offered comprehensive wellness-focused medical assessments as part of routine SBHC clinical practice, and enhanced the MMCC afterschool group-based provision of services to provide healthy lifestyle behavior change education, cooking classes, and daily physical activity opportunities (Table 1). The program incorporated onsite Wellness in the Schools (WITS) family support to cook healthy meals and the Prevention Intervention Research Center (PIRC) expertise to incorporate youth resilience and YD concepts into the program. With a USDA-funded school food program, the PS/MS-95 School is mandated to have a Wellness Council where teachers, students, parents, MSHP Community Health Organizer, and administrators collaborate to support wellness programming at the school. In partnership with the Wellness Council, *B'N Fit POWER* staff learned about the need for and interest in healthy lifestyle programming, barriers to enrollment and program engagement, and outcomes of interest.

Implementation entailed a two-year planning phase, where the B'N Fit Director met separately with the key stakeholders. The first-year quarterly meetings with the MMCC administration focused on afterschool recruitment and engagement strategies, collection of screening and program attendance data, reducing potential stigma related to a school-based obesity intervention, and the structure and curriculum content of the afterschool program. Quarterly meetings with the MSHP staff focused on developing clinical assessment tools, provision of clinical services, and collection of clinical data. Quarterly meetings with the PIRC staff focused on the design of the program evaluation and outcome metrics, strategies for reducing stigma, and strategies for incorporation of YD elements into the afterschool program curriculum. An initial meeting with the Principal of PS/MS-95 was also conducted to obtain permission to pilot the program in the school. During the second year, separate meetings with each of the MMCC, MSHP, and PIRC stakeholders occurred monthly to discuss the implementation strategies in more detail, and meetings with the principal occurred quarterly. Monthly meetings with the School Wellness Council began in the latter part of the second year in preparation for a fall program start, and the partnership with the WITS staff, MSHP Community Health organizer, students, and teachers focuses on engaging parents during biannual Family Fun Fitness Nights, developing efficient protocols for acquisition of outcome metrics, and supporting a healthy lifestyle culture at the school.

### 2.2. Setting/Context

The Bronx, which is the poorest urban county in the United States and least healthy county in New York State, has high ethnic diversity (54% Hispanic and 43% African-American). Childhood poverty is 43%, and school absenteeism is high, as is the prevalence of obesity [1, 40, 41]. Data from the New York City Department of Education indicate that 70.6% of PS-95 students live in households that are below 130% of the poverty level [42], and nearly half (46.9%) of all Bronx residents report feeling at risk of becoming homeless [43]. The low SES of teens living in the Bronx limits their exposure to opportunities that can make a difference in their lives and reduce health disparities compared with other teens [44]. Despite these immense barriers to attaining good health in the Bronx, the long-term presence and commitment to providing onsite health and afterschool services by the MSHP and MMCC, respectively, along with the efforts of the principal and onsite stakeholders to provide and sustain established quality health education for its students qualifies this school to implement the services of *B'N Fit POWER* with an opportunity to create an integrated program to address these disparities.

### 2.3. Selecting Intervention Outcome Metrics

Program outcome metrics were selected if they could be commonly utilized by all partners and if they could be accurate, credible, and reproducible measures of progress. Primary outcomes included the following: (1) Height and weight determinations done at the SBHC using a Scaletronix® scale as per routine clinic assessment. (2) The NYC Fitnessgram: the current measure of fitness for all NYC public school students (approximately 85% of NYC public school students have BMI measured annually via the NYC FitnessGram) [45]. Students are assessed in four fitness areas, cardiovascular fitness or aerobic capacity, muscle strength, muscular endurance, and flexibility, and scores are evaluated against criterion-based “Healthy Fitness Zone®” standards that indicate the level of fitness necessary for health. (3) The 51-item *B'N Fit POWER* survey data, which include assessment of the *7 Target Behaviors*, nutrition knowledge, and outcome expectancy. The *7 Target Behaviors*, which were developed, following the Expert Committee Recommendations, USDA MyPlate dietary guidelines, and NHLBI sleep recommendations [46–48], are detailed in Table 2. Secondary outcomes include improvements in cardiovascular and diabetes risk as measured by routinely collected markers of cardiovascular disease (LDL, triglycerides, and HDL) and insulin resistance (HbA1C). Additional routinely collected outcomes include school attendance and grades, attendance at afterschool sessions tracked using the MMCC afterschool NYC Department of Youth and Community Services (DYCD) database.

### 2.4. Selecting Implementation Outcome Metrics

Our implementation outcome metrics employed a hybrid strategy that combined elements of the RE-AIM framework and a Wellness Adaptation of the HIV cascade. The RE-AIM approach to evaluating the public health impact of health promotion interventions is based on 5 factors: reach, efficacy, adoption, implementation, and maintenance [49]. The underlying principle of the framework is that, beyond program effectiveness, the impact of system-based, community-based, or public health interventions relates to the contribution of each evaluative dimension, but a majority of studies fail to equally discuss all elements of the model [50–52]. Additionally, we adapted the HIV Cascade, which has been used as a conceptual framework for evaluating HIV treatment programs and represents population level representation of involvement in sequential steps of HIV treatment [53]. The hybrid strategy or “Wellness Cascade” evaluates the successive steps of our program implementation by establishing (1) the proportion of students that are available to be screened; (2) the proportion of students that are diagnosed with overweight and obesity; (3) the proportion that are recruited and enrolled in the program; (4) the proportion that are compliant with program requirements and thus engage in treatment; (5) the proportion that are retained in the program and thus complete the treatment; and (6) the proportion with a successful outcome (Table 3)

### 2.5. Intervention Evaluation

To evaluate the feasibility of implementing the program and secondarily to evaluate its effectiveness, we designed a quasiexperimental trial of *B'N Fit POWER* to compare students receiving *B'N Fit POWER* to students who chose not to enroll in the program and receive standard of care to address the following specific aims: (1) to determine whether *B'N Fit POWER* is effective in improving fitness, healthy weight attainment, and 7 target behaviors; (2) to assess the impact of moving the B'N Fit program into a school setting on participant engagement; and (3) to assess the mediating pathways associated with program effects. Following an initial health screening, students voluntarily enrolled in *B'N Fit POWER* (Group 1) were to receive comprehensive medical assessments at the MSHP integrated with MMCC afterschool programming that incorporates a curriculum focusing on *7 Target Behaviors* during weekly leadership sessions and daily physical activity. The comparison group consisting of all other students not enrolling in *B'N Fit POWER* (Group 2) would receive the standard of care (standard MSHP and MMCC afterschool program).

Using the MMCC Afterschool program DYCD database, an opt-out letter was sent home to all MMCC afterschool program participants who would be incoming middle school students in the 2016/2017 school year inviting them to participate in a health assessment in the spring of 2016. The screening consisted of the following: (1) height and weight assessments were done in the MSHP SBHC, and BMI was calculated by the PI and (2) NYC Fitnessgram and *B'N Fit POWER* survey data were conducted by a physical activity specialist and youth leaders during afterschool program physical activity and leadership sessions, respectively. Students then received printed materials supporting healthy diet and physical activity behaviors. In addition, to improve access for youths who were not in afterschool, BMIs of all students registered in the MSHP SBHC were reviewed as part of the electronic medical record (EMR) review. These students, if not already registered in the afterschool program, did not undergo the screening. Although open to all, students whose BMI ≥ 85th percentile for sex and age were actively recruited and approached about enrolling in the program during the course of routine interactions with both MMCC and MSHP staff as well as via telephone outreach by the B'N Fit Staff. Additionally, distribution of flyers in the SBHC and to MMCC settings and a family information session promoted awareness about the program at the school. Emphasis was placed on explaining the physical activity and health benefits of participating in *B'N Fit POWER* rather than focusing on BMI and the need for weight loss. The target enrollment was to recruit at least 85% of program participants to have a BMI ≥ 85th percentile.

Once screened as per the *B'N Fit POWER* protocol, students were invited to enroll in *B'N Fit POWER* if they were 11 to 14 years, going to be in the 6th–8th grade in the fall of 2016, able to register in both the MMCC afterschool program, and in the MSHP SBHC, and a parent or guardian was available in person or by phone for clinic visits. Students were excluded if they had a mental illness that would render them incapable of consenting for the research or complying with the *B'N Fit POWER* afterschool program protocol or had medical problems that made it unsafe for them to participate in the program. The study was approved by the Institutional Review Board. Parents signed informed consent, and HIPAA authorization and participants provided assent at their initial SBHC visit. Once enrolled in *B'N Fit POWER*, students underwent the 60-minute baseline SBHC-adapted *B'N Fit POWER* wellness assessment during the summer months. The rationale for conducting the baseline assessment over the summer was to limit the time taken out of class for the initial assessment during the school year. During the initial assessment, a brief 2-page weight history questionnaire and the *7 Target Behavior* questionnaire would be filled out by the patient and entered into the EMR that is on the Epic (onsite EMR) platform [55] by the licensed practical nurse (LPN). The parent would complete the Pediatric Symptom Checklist, a validated 17-item behavioral screen addressing internalization, externalization, and attentional domains, to also be entered into the Epic platform by the LPN. The medical provider would then review the questionnaires and conduct a brief nutrition assessment (including a 24-hour diet recall), focused physical exam, including BP assessment, and depending on their BMI and BP, the student would undergo lab evaluations that were agreed upon by MSHP staff based on cost and feasibility of obtaining in the SBHC (Table 2). As per SBHC protocol, students received onsite mental health services if the PSC17 was positive in one or more domains. At a second 15-minute follow-up visit, the provider reviewed lab work and an Epic generated 1-page treatment plan summarizing comorbidities, target behavior, and weight goal and plan to achieve target changes. Following the initial assessment, students would return every 6 to 8 weeks for a total of four additional follow-up visits to monitor their progress with target changes.

Following their initial assessment, students participated in the MMCC afterschool program which provides afterschool programming for elementary and middle school students conducted from 3 to 6 pm daily from Monday through Friday throughout the school year. Similar to the other students in the afterschool program, who are required to attend 3 hours of Leadership each week, students enrolled in B'N Fit had the option of creating their own schedule with some minimum requirements: (1) attend three hours of *B'N Fit POWER* leadership sessions which included the *B'N Fit POWER* leadership curriculum, cooking class, and core physical activity education; and (2) attend at least 5 hours of physical activity programming each week. Attendance at all afterschool activities was tracked as per routine.

### 2.6. Implementation Evaluation

To evaluate the implementation feasibility, we collaboratively developed a strategy to integrate program metrics into the SBHC, MMCC afterschool, and school work-flows of onsite community partners using a Plan-Do-Study-Act (PDSA) cycle that followed the school year schedule [56]. Program improvement planning was done over the summer and implemented throughout the school year. MMCC collected attendance data and SBHC collected follow-up BMI and target behavior data throughout the school year. Outcomes were analyzed over the summer to inform follow-up improvements for the fall. Entry of anthropometric, lab, and target behaviors into Epic as part of the routine clinic visit aligned with current MSHP SBHC provider practice and provided a built-in sustainable method for tracking program outcomes. Further, attendance at *B'N Fit POWER* afterschool sessions was taken as per routine in the MMCC program supporting a sustainable method for tracking attendance and compliance with *B'N Fit POWER* leadership and physical activity requirements and supported a built-in structure for the program evaluation. Existing monthly onsite Wellness Council meetings provided a forum for discussing progress at each stage of the PDSA cycle with all stakeholders.

In the following results section, we describe the strategies used to maximize and optimize the screening for overweight and obesity, establish the feasibility of diagnosing students with overweight and obesity, facilitate enrollment and linkage to care, and program implementation. Results related to efficacy, receiving and completing therapy, and attainment of successful outcomes will be discussed elsewhere.

## 3. Implementation Results

### 3.1. Screening, Diagnosis, and Enrollment into the Program

**Figure 2:**
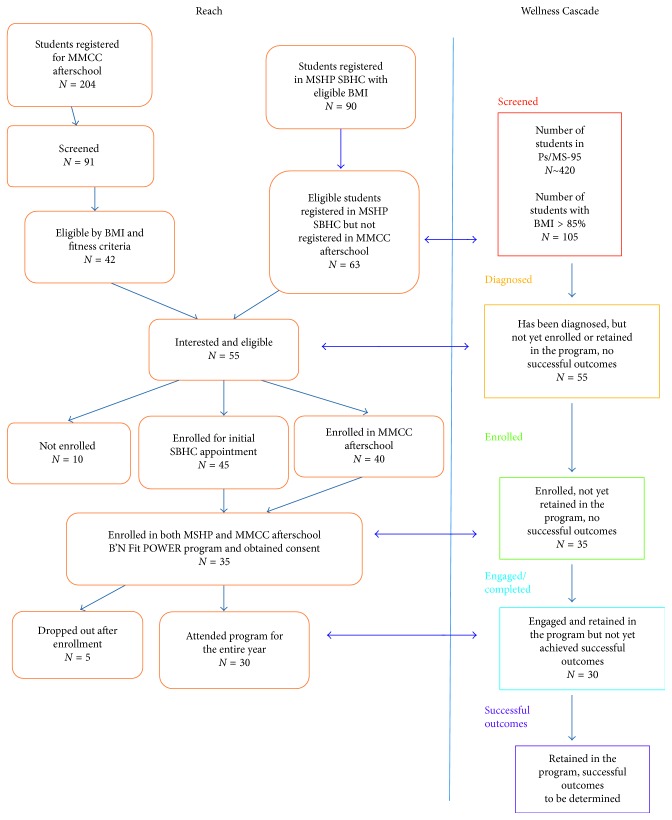
Implementation evaluation based on RE-AIM and Wellness Cascade.

We recruited students from the SBHC and MMCC afterschool programs. In the spring of 2016, 204 students in 5th–7th grade were registered in afterschool and 91 students completed screening during afterschool program hours, and of these, 42 (46%) met BMI criteria (BMI ≥ 85th percentile). An additional 63 students identified from the SBHC EMR review met BMI criteria. Of approximately 420 middle school students attending the school, we were able to obtain screening BMI data on 154 youths (37%), and of these, 105 met the BMI criteria of having a BMI ≥ 85th percentile. Of these students diagnosed with overweight or obesity, 55 (52%) were interested in the program (45 referred through MMCC; 10 referred through SBHC) (Figure 2). Of these, 35 registered in both SBHC and MMCC and completed signed parent consent and child assent. Of the 35 youths with IRB consent, 30 (86%) had a BMI ≥ 85 percentile for sex and age (Figure 2).

### 3.2. Process Evaluation of Screening, Diagnosis, and Enrollment Procedures

Our process evaluation identified challenges that included the following: (1) Identifying students from the afterschool program who achieved a low screening yield (91/420 students) and recruiting via the EMR to identify that elevated BMI did not align with efforts to reduce stigma and demedicalize the program for youths not already in the afterschool program. (2) Inadequate B'N Fit staff training limited opportunities to organizing training sessions; per diem youth leader staff had time committed to other activities. (3) The questionnaire was perceived as lengthy and difficult for students and MMCC staff to understand and complete. In addition, offsite PI obtained weight and height assessments during afterschool hours (as SBHC staff were unavailable during this time), and telephone outreach by offsite B'N Fit staff were unsustainable protocols. (4) The lack of a formalized communication protocol between SBHC (who worked during the school day) and MMCC afterschool staff (who worked during afterschool hours) delayed students enrolling through MMCC getting into the SBHC and delayed students enrolling through the SBHC getting into MMCC.

The process evaluation was used to develop new strategies for subsequent screenings (Table 4). Stakeholder input, including the school principal, MMCC staff, onsite Educational Consultant and Physical Education Faculty, and technical assistance from the HRSA granting agency, resulted in plans for (1) screening during the school day in conjunction with the schoolwide NYC Fitnessgram (weight and height data) done by physical education teachers and (2) administering the questionnaire, which was shortened to 25 items and computerized, during health class required for all students. These strategies address the limitations of initial screenings related to ensuring reduction of stigma and demedicalizing the program, while increasing the number of youths that can be fully screened potentially reaching over 400 middle school students annually.

Conducting the screening during the school day as proposed has been approved by the IRB and is currently being initiated. A bidirectional referral system involving the SBHC and the MMCC afterschool program staff was developed to ensure that overweight or obese youths, who might not otherwise join the *B'N Fit POWER* program, were diagnosed and enrolled in both settings. Utilizing the schoolwide NYC Fitnessgram data to identify all middle school students with a BMI > the 85th percentile, the PI provided a list to the MMCC staff who focused on recruiting students enrolled in afterschool and the SBHC staff who focused on all others. As students enrolled for the afterschool program in the fall, MMCC staff spoke individually with students and their parents diagnosed with a BMI > 85th percentile about registering in both MMCC and SBHC for enrollment in *B'N Fit POWER*. The SBHC staff conducted Brief Health Assessments (BHA) (which are routine visits unique to the SBHC intended to connect students to a medical home) on students diagnosed with a BMI > 85th percentile and would similarly speak with students and their parents about enrollment in the program. A per diem program monitor was hired by the MMCC afterschool program in the fall of September 2017 to create a protocol to facilitate these bidirectional referrals and as of the writing of this protocol, 35 youths have enrolled in both the SBHC and afterschool program with 11 referred through the SBHC and 24 referred through the afterschool program.

### 3.3. Engagement in the Clinical and Afterschool Aspects of the Program

Of the 35 youths with IRB consent, 30 engaged in the program and attended elements of the program throughout the school year. To address stigma, youths in *B'N Fit POWER*, similar to the other students in the afterschool program, had the option of creating their own schedule with some minimum requirements. All students in the afterschool were assigned to leadership groups which consist of approximately 15 to 20 youths who stay together for the entire year. The structure of the MMCC afterschool program allowed for a seamless integration of the *B'N Fit POWER* participants into the program with other afterschool participants, particularly because there is overlap with many of the nonleadership classes and physical activity opportunities. The integration of *B'N Fit POWER* into the existing afterschool structure required no additional need to enter *B'N Fit POWER* attendance into a separate database.

### 3.4. Process Evaluation of Engagement in the Clinical and Afterschool Aspects of the Program

Challenges affecting implementation of a comprehensive weight management assessment in the busy SBHC and ensuring student engagement into the program included the following: (1) Lack of parental availability to obtain written consent during the school day resulted in research staff spending an unsustainable amount of time and effort to meet parents onsite and even offsite to ensure that written consents were completed. (2) Limited availability of students and families to undergo clinical assessments over the summer due to family vacations and lack of availability resulted in only 13 students undergoing their initial clinical assessments during the summer months. (3) The 60-minute initial visit was lengthy and difficult to implement during the fall school schedule during busy clinic schedule. (4) Lack of highly trained and specialized nutritionists onsite to support healthy lifestyle education placed the burden of nutrition education on the onsite medical provider, who often had little formal nutrition education and limited time to see patients. (5) While we had initially planned to do four additional follow-up visits throughout the year, the length of time it took to complete all initial visits due to high staff turnover and hospital-wide implementation of Epic in the fall requiring extensive offsite employee training (all initial visits were completed in January of 2017) made this impractical to achieve.

The process evaluation was used to develop new strategies for subsequent clinic visits to ensure engagement into the program (Table 5). In discussions with parents and staff, the standard requirement for registering in both the SBHC and the afterschool program made the additional requirement of written research consent a barrier to completion. In an effort to streamline the consent process, in June of 2017, we obtained an amendment to the IRB to allow for verbal parental consent in combination with written child assent to facilitate study enrollment. To reduce time spent in the clinic, the initial visit was divided into two 20-minute Brief Health Assessments. The questionnaires are now being sent home and brought in completed. During the initial assessment, the history and physical exam are completed and target behaviors are identified. Labs are done during the week, and then during second 20-minute visit, the labs are discussed, comorbid conditions are reviewed, and target weight and target behaviors are discussed with 1-page treatment plan. To support healthy lifestyle education, one-page educational sheets reflecting practical approaches to making specific target behavior changes have been developed, and they increase the efficiency of providers. In consultation with MSHP staff, it was decided that each student would undergo only two additional follow-up visits, one in early winter and one in late spring. With this adjustment to the protocol, we were able to complete a first and second follow-up visit on 30 youths resulting in an 86% clinic retention rate. With the integration of the above changes, 27 students have completed their consent and baseline SBHC visit so far for the 2017/2018 school year.

The main challenges affecting the implementation of *B'N Fit POWER* into the afterschool setting included the following: (1) Lack of highly specialized and trained staff to teach the *B'N Fit POWER* comprehensive healthy lifestyle curriculum during leadership sessions and the need for youth leader training. (2) Importantly, the clinician designed *B'N Fit POWER* curriculum lessons were often lengthy and not well understood by youth leaders, who typically had a high school education and limited prior nutrition and healthy lifestyle education or experience. (3) Further, recipes contained within the *B'N Fit POWER curriculum* were also not well accepted by staff and youths because they used ingredients that were generally not readily available in the neighborhood surrounding the school. (4) Finally, the busy afterschool setting made it difficult for staff to receive training related to the curriculum and adapt recipes to better reflect cultural preferences and neighborhood grocery availability.

The process evaluation was used to develop new strategies to support student engagement into the program implementation for the 2017/2018 school year (Table 5). With much discussion and feedback from the onsite staff, an additional role for the Program Monitor, hired by MMCC, was to take on the role of serving as the MMCC education specialist to review curriculum. The curriculum has been reformatted to the lesson plan format already being used by the youth leaders; lesson plans have been edited to ensure that the content is clear, relatable, and relevant, and the Program Monitor trains youth leaders in advance of sessions to ensure that the curriculum remained relevant and engaging, and the per diem cooking specialist, who is familiar with the types of foods available in the community, has altered recipes to support the successful implementation of the recipes. We have also partnered with WITS to incorporate Department of Education alternative menu approved recipes into the cooking curriculum reinforcing concepts taught during the school day.

## 4. Discussion

To address health disparities in our community, we successfully engaged with a school and its onsite stakeholders and completed our first year of implementing *B'N Fit POWER*. We initiated protocols to support engagement into the school, piloted implementation of program components, and utilized our Wellness Cascade strategy to evaluate four successive steps of our program implementation corresponding to screening, diagnosis, enrollment, and engagement. While we have met a number of challenges related to the implementation process, we have developed strategies moving forward for (1) increasing the proportion of students screened from focusing on about a quarter of the middle school population to the entire middle school population; (2) ensuring that there is a mechanism for communicating with all students whose BMI > 85th percentile that there is healthy lifestyle programming available at their school and supporting linkage to care; and (3) supporting the feasibility of integrating clinical and afterschool components of the program into routine services offered at the school.

### 4.1. Initial Lessons Learned

The overarching lesson learned during the pilot implementation of the *B'N Fit POWER* program was that stakeholder collaboration provided the foundation for developing *B'N Fit POWER*, which included components of both successful hospital-based intensive and school-based interventions. These components included comprehensive clinical services, afterschool school program with participatory/hands on skill building student activities to teach strategies for improving dietary intake, increased physical activity, and behavioral techniques (including self-monitoring, goal setting, or coping skills) and to provide a daily dose of physical activity. *B'N Fit POWER* also included teacher training, providing a program for a duration of a year or longer, tailoring for cultural relevance, and involving parents as essential stakeholders [17, 35, 36]. The shared stakeholder responsibility facilitated delivering this content in a consistent and effective manner that ultimately results in successful implementation and sustainment of such a program. To do so, we learned how important it was for stakeholders to trust one another, the importance of giving the appropriate time and support to implement the program, particularly in our setting with few resources and limited funding, and the importance of acknowledging student stakeholder identified barriers, namely, the stigma that accompanies obesity, to program enrollment.

The B'N Fit staff has begun to earn the trust of the various stakeholders, especially with respect to “doing research” on *B'N Fit POWER* by focusing on collaborative decision-making approach [57, 58]. We learned that to gain trust we needed to consistently communicate that we valued stakeholder input and that we shared a common goal of improving the health of the students attending the school. We strove to work together as a coordinated team to accelerate the attainment of these goals during multiple stakeholder meetings in the two years leading up to the pilot intervention. Although the B'N Fit staff had extensive experience with designing, implementing, and evaluating a hospital and community-based weight management program, we acknowledged that onsite staff had more extensive experience serving large numbers of youths in their respective areas of expertise. The B'N Fit staff also joined the school-initiated School Wellness Council that meets on a monthly basis to gain a better understanding of the school health mission, current activities that promote health, and the current stakeholder strengths and resources and barriers to implementing healthy initiatives. While respecting the requirements governing the individual school, clinic, afterschool systems, and evaluation requirements, our collective priority was to ensure that the needs of the students guided our common efforts. We were thus able to streamline the screening and diagnosis protocols and align them with existing school fitness screening protocols and refine enrollment, and engagement processes by limiting time out of class to complete clinical assessments, utilize existing afterschool attendance data, and adapt the programming to be consistent with existing leadership, literacy, and STEM (science, technology, engineering, and math) requirements [59] and activities. Implementation of the PDSA evaluation cycle enabled the integration of *B'N Fit POWER* programming within the school calendar and work plans, for example, shared planning and program execution, working together to develop policies and procedures that systematized the coordination of services. Finally, support for the interprofessional team via the development of a training infrastructure facilitated more efficient use of resources and a further building of trust thereby also integrating staff engagement and empowerment into the program resulting in improved service for our target audience.

Drawing on the widely used SMART (specific, measurable, attainable, relevant or realistic, and time-bound) goal setting [60] approach enabled us to engage students and personalize setting lifestyle change goals as well as for setting program goals. We learned that careful planning was required to come up with a set number of specific annual goals for each aspect of the program and that these goals were measureable, attainable, relevant, and could be accomplished during the school year. During this process, we realized that it was imperative to consider the student's community context and stakeholder time constraints and barriers to implementation. Understanding that the Bronx is the nation's poorest urban county and least healthy county in NY State and that youths and families serviced by this program were at high risk for health disparities meant that there were high parental unemployment rates, limited financial resources, limited access to healthful foods, transportation for their children, or afterschool care for younger siblings at home. We learned that, for these youths, their families, and the staff that service these youths, minimizing the number of steps needed to engage youths in the program would support screening, diagnosis, linkage to care, and participation in the program and that the implementation of the program would need to occur over time with multiple brief actionable steps via PDSA cycles for each level of the program implementation. There is an underlying urgency to make large changes quickly to improve the health statistics in the Bronx, yet resources are sorely lacking. Applying lessons learned both from a decade of working with adolescents who are struggling to make healthy lifestyle changes and from the first year of implementing *B'N Fit POWER*, we learned that if we are a patient and focus on success with smaller goals, over time, we will empower the entire team to move forward to achieve larger goals and continue to make sustainable changes.

A final lesson learned related to acknowledging student stakeholder identified barriers, namely the stigma that accompanies obesity, to program enrollment. Our prior experience in B'N Fit with provider referrals based on BMI cutoffs resulted in youths enrolling in the B'N Fit program at the urging of their family members or provider, with a majority (70%) being in either the precontemplation or contemplation stage of change related to their interest in attending the program and making lifestyle changes; [22] most did not tell friends they attended a weight management program. While prioritizing eliminating stigma by opening enrollment to all students in the school, we acknowledged that high-risk youths may not volunteer for a popular afterschool programming expressly because they feel self-conscious, are concerned that they cannot keep up with activities and may be bullied about their physical appearance or physical limitations related to their weight, or may have other personal, familial, or social barriers to participation [61–63]. Generalized afterschool screening and open program access for all students in the afterschool program minimized stigma, as evidenced by the popularity of the program in its first year, while targeting at least 85% of the enrollees with a BMI ≥ 85th percentile. This strategy ensured adequate access to healthy lifestyle programming for high-risk youths who might not ordinarily access such a program and who would likely benefit the most from its services. Broadening the screening to include the entire school will serve to further reduce the stigma by ensuring that all students, including those not already enrolled in the existing afterschool program, undergo the same screening process. Expanding the number of students that can enroll in the afterschool *B'N Fit POWER* in the future will further increase the proportion of at risk students who have access to this program.

### 4.2. Limitations

Transferring the acquisition of the data from the afterschool setting to the school day setting has limitations related to the timing and accuracy of the data. Afterschool screening Fitnessgram, height and weight, and questionnaire data were obtained in April, May, and June annually, which was used for the pre- and postprogram evaluation. However, screening data are typically collected as per school protocol during physical education and health education classes from October through April. Thus, our approach for increasing the reach and efficiency for screening students does not necessarily align with a baseline and follow-up evaluation. Although the timing of the data collection conducted by the school may be viewed as a limitation, an assessment of overall school health and the impact of the interventions can be evaluated on an annual basis, and the use of this method already systematized by the school ensures sustainability of the screening process. An additional limitation relates to the use of a quasiexperimental trial to compare students receiving *B'N Fit POWER* to those students at the same school not in the program and who receive standard of care. The lack of the use of a RCT introduces selection bias, and the availability of overlapping programming activities introduces contamination thus affecting the robustness of the evaluation. Taking into consideration, however, the need for efficient use of resources and building stakeholder trust to prioritize a sustainable service for the youths, it has become clear that establishing the feasibility and acceptability of implementing the program and designing a realistic evaluation strategy is a necessary first step to designing an effectiveness trial for the program. While we conducted a process evaluation for engagement into care, we did not describe results related to actual number of students attending various components of the program as a measure of engagement and program compliance. Although attendance data were available, it was not readily accessible from the DYCD database, and the development of a protocol for accessing this data on a regular basis is necessary to track afterschool program attendance and compliance and is currently under development during the second year of the program implementation.

### 4.3. Next Steps

Moving forward the identification of individual stakeholders who will take on the role of all onsite training staff, implementation of quality assurance and quality control procedures, as well as the establishment of protocols to ensure collaboration among interprofessional teams will facilitate the clinical-community integration process. Utilization of tools for measuring the interprofessional collaboration and refinement of the Wellness Cascade strategy for measuring the impact of the collaboration will strengthen the integration process. Results related to engagement and completion of the program and attainment of successful outcomes will be reported in terms of the Wellness Cascade and further inform future implementation efforts.

## Figures and Tables

**Table 1:** Contributing stakeholders and their role in the *B'N Fit POWER* implementation.

Stakeholders	Description	Staffing	Implementation role in *B'N Fit POWER*
Bronx Nutrition And Fitness Initiative For Teens	*Program activities*: Adolescent Weight Management Program operated out of the Children's Hospital at Montefiore	(i) 0.15 FTE Project Director^∗^ (ii) 0.5 FTE POWER Program Coordinator^∗^ (iii) Student volunteers	(i) Provision of guidelines for screening, clinical evaluation, treatment plans, and afterschool requirements (ii) Development of program metrics (iii) Integration of stakeholder efforts (iv) Conducts MMCC afterschool staff trainings to support screening process (v) Conducts height and weight screenings

Mosholu Montefiore Community Center (MMCC)	*Program activities*: operates afterschool (3–6 pm) programs in 15 sites: (i) Recreation and physical activity opportunities (ii) Educational support (iii) Enrichment and socialization (iv) Youth leadership	(i) In-kind program director (ii) In-kind youth leaders ×4 (iii) In-kind administrative staff (iv) Per diem cooking specialist^∗^ (v) Per diem exercise specialist^∗^	(i) Supports community-led recruitment (ii) Provides afterschool group-based healthy lifestyle behavior education, cooking and gardening classes, and daily physical activity opportunities (iii) Integrates *B'N Fit POWER* into existing afterschool program structure

The Montefiore School Health Program (MSHP)	*Program activities*: operates during the school day (8 am–3 pm); provides onsite coordinated primary and preventive health care in 25 locations (i) Delivers medical, mental health, dental, and community health services	(i) In-kind primary care MD/NP (ii) In-kind social worker (iii) In-kind licensed practical nurse (iv) In-kind, community health organizer (CHO) (v) In-kind receptionist (vi) In-kind medical director	(i) Develops efficient clinic protocols (ii) Conducts comprehensive wellness medical assessments, ordering standard lab set, generates 1-page treatment plan, and supports healthy lifestyle education as part of the routine onsite clinical practice (iii) Conducts follow-up visits as per routine clinical practice to monitor progress

PS/MS-95 School And Wellness Council	*Governance activities*: USDA-funded school food program, mandated to have an onsite Wellness Council	(i) School principal (ii) MSHP CHO (iii) Education consultant (iv) PTA president (v) WITS staff (vi) Students and parents	(i) Facilitates understanding of existing healthy lifestyle programming (ii) Incorporates target behavior concepts into school wellness activities (iii) Establishes outcomes of interest

Prevention Intervention Research Center (PIRC)	*Research evaluation activities*: develop and test interventions to prevent mental health problems in children and youths with chronic health conditions	(i) In-kind Director (ii) In-kind developmental psychologist (iii) Program staff	(i) Supports program evaluation (ii) Incorporates YD concepts into the program curriculum

Wellness in the Schools (WITS)	*Program activities*: national nonprofit that teaches kids healthy habits to learn and live better	(i) In-kind onsite chefs	(i) Supports healthy menus during cooking and family events

Students and families	Students attend school, B'N Fit afterschool, and on wellness council, parents serve on PTA	(i) Student volunteers (ii) Parent volunteers	(i) Informs need for and interest in program (ii) Identifies barriers to enrollment, program engagement, and outcomes of interest

^∗^Staff funded specifically for *B'N Fit POWER*, and all others staffing in-kind or volunteers.

**Table 2:** Intervention outcome metrics.

Clinical assessment	

Anthropometrics	Height and weight
Vitals	Blood pressure
Laboratory evaluation	(1) If BMI < 85th percentile—no additional labs
	(2) If BMI ≥ 85th percentile—lipids and HbA1c

Fitness assessment	

NYC FitnessGram	(1) Cardiovascular fitness
	(2) Aerobic capacity
	(3) Muscle strength
	(4) Muscular endurance
	(5) Flexibility

Behavioral outcomes: 51-item B'N Fit survey	

*Subscales*	*Number of items*
7 target behaviors	14 items in questionnaire:
	(1) Eat breakfast and lunch daily
	(2) Eat 2-3 servings of fruits a day
	(3) Eat 3–6 servings of vegetables a day
	(4) Drink 8 cups of water daily/limit sugary drinks to ≤1 cup daily
	(5) Sleep at least 8 hours a night
	(6) Get at least an hour of physical activity daily
	(7) Eat unhealthy snack foods or fast foods no more than weekly
Self-efficacy, outcome expectancy, school attendance and grades, nutrition knowledge, and behaviors	Total 37 items

Other	

Afterschool attendance	Daily attendance at MMCC afterschool program

**Table 3:** Implementation outcome metrics: Wellness Cascade.

Wellness cascade steps	Aim at each step
(1) Proportion of students *screened*	Screening all students in the school annually
(2) Proportion of students *diagnosed* with overweight or obesity	Identifying all students in the school with a BMI ≥ 85th percentile annually
(3) Proportion of students recruited and *enrolled* in the program	Recruitment aim of having at least 85% of the participants recruited annually with BMI ≥ 85th percentile
(4) Proportion of students that *engaged in treatment* and compliant with program	Aim for students to attend all clinic visits and at least 75% of afterschool sessions during the year
(5) Proportion that are retained in the program and thus *completed the treatment*	Aim for at least 75% of students retained in the program for the entire school year annually
(6) Proportion of youths that attain a *successful outcome*	Aim for at least 50% of participating youths achieve a BMI *z*-score reduction of 0.2 defined as clinically important change [54]. Aim for a 25% improvement in the attainment of 7 target behaviors

**Table 4:** *B'N Fit POWER* integration of screening, diagnosis, and enrollment procedures.

Procedures	Existing PS-95 structure	*B'N Fit POWER* intervention at PS-95	Implementation challenges	Solutions to challenges
Screening for students with BMI ≥ 85th percentile	(i) Schoolwide NYC fitnessgram screening not routinely accessed as screening tool by MMCC or SBHC staff (ii) No routine screen by MMCC/SBHC staff	(i) Opt-out letter sent home to all students in 5th–7th grade registered in afterschool (ii) Afterschool program screening: (1) Height/weight (PI) (2) Fitnessgram (physical activity specialist) (3) *B'N Fit POWER* survey (youth leaders) (4) Healthy lifestyle handout (iii) EMR review of students in SBHC	(i) EMR identification of students with BMI > 85th percentile did not reduce stigma (ii) Low numbers screened in afterschool program (iii) Inadequate B'N Fit staff training of onsite MMCC staff (iv) Lengthy questionnaires difficult to understand and complete (v) Weight and height assessments by offsite PI unsustainable	(i) Consent letter sent to all students in school (ii) Schoolwide NYC fitnessgram data obtained by physical Education staff (iii) Access for all students (iv) B'N Fit staff training of educational consultant to train teachers to conduct questionnaire screening (v) Questionnaire shortened to 25 online items

Diagnosis	(i) No routine protocol for communicating overweight or obesity to at-risk youths (ii) SBHC BHA not always focused on screening for BMI ≥ 85th percentile	(i) Students with BMI ≥ 85th percentile recruited during routine interactions with MMCC and MSHP (ii) Emphasis was placed on explaining physical activity and health benefits of the program (iii) Telephone outreach by B'N Fit staff (iv) Distribution of flyers (v) Family information session	(i) Telephone outreach by B'N Fit staff unsustainable (ii) Low attendance at family information session	(i) NYC fitnessgram height and weight data provide shared recruitment list (ii) MMCC focus outreach on students in afterschool (iii) SBHC focus outreach on students not in afterschool

Enrollment	(i) Self-referral to SBHC (ii) Self-referral to MMCC afterschool program	(i) Self-referrals (ii) Telephone outreach (iii) Flyer distribution (iv) Information session (v) Target 85% of enrollees with BMI ≥ 85th percentile	(i) Delays in getting students enrolled in both MMCC afterschool program and SBHC related to interinstitutional communication challenges	(i) Bidirectional referrals (ii) Hired program monitor for referrals protocol implementation

BHA: Brief Health Assessment; PI: principal investigator; EMR: electronic medical record.

**Table 5:** *B'N Fit POWER* integration of SBHC visits and afterschool program components.

Procedures	Existing PS-95 structure	*B'N Fit POWER* intervention at PS-95	Implementation challenges	Solutions to challenges
SBHC baseline clinical assessment and follow-up visit	(i) BHA not consistently focused on screening for BMI ≥ 85th percentile (ii) No protocol for routine treatment and follow-up of youths with elevated BMI	(i) Obtain written parental consent and child assent (ii) 60-minute initial visit (iii) Patient fills out 2-page weight history, *7 Target Behavior*, and *PSC-17* questionnaires, (iv) LPN (1) Height, weight, BP (v) Medical provider: (1) Questionnaire review (2) Nutrition assessment (3) Focused PE (4) Orders labs (vi) MHP referral per routine (vii) 2nd 15-minute visit to review labs and treatment plan (viii) 4 follow-up visits to review behavior and weight goals	(i) Difficulty obtaining written parental consent (ii) Lengthy 60-minute visit difficult to implement during school schedule (iii) Lack of onsite specialized nutritionists to support healthy lifestyle education (iv) Busy clinic (v) Length of time to complete the initial visits made 4 follow-up visits impractical to achieve	(i) Obtain verbal consents from parents rather than written consent (ii) Split initial 60-minute visit into two 20-minute BHA visits: (iii) Visit 1: complete questionnaires, PE, and labs (iv) Visit 2: review lab work, and 1-page treatment plan, and target behavior education (v) Limit follow-up visits to two additional visits (vi) Completed repeat labs on those students with abnormal labs at initial visit

MMCC Afterschool Program	(i) From 3 to 6 pm daily (M-F) during year (ii) Includes DYCD mandated three hours of leadership (iii) Optional activities (iv) Routinely collect daily attendance (v) No routine curriculum for healthy lifestyle education	(i) DYCD mandated leadership includes the following: (1) *B'N Fit POWER* leadership curriculum (2) Cooking class (3) Gardening (ii) At least 5 hours of physical activity programming weekly (iii) *B'N Fit POWER* participants may sign up for any other optional activity available to the other students in the afterschool program	(i) The need for youth leader training (ii) *B'N Fit POWER* curriculum lessons were often lengthy and not well understood by youth leaders (iii) Recipes in the *B'N Fit POWER curriculum* not well accepted because ingredients not readily available in the neighborhood (iv) Busy afterschool setting	(i) Program Monitor serves as the education specialist and has reformatted and revised lesson plans to ensure clear, relatable, and relevant content (ii) Program Monitor trains youth leaders in advance of sessions (iii) Cooking specialist and WITS chefs altered recipes to better align with local community food availability

BHA: Brief Health Assessment; LPN: licensed practical nurse; MHP: mental health provider; PE: physical education; WITS: Wellness in the Schools.

## Abbreviations

B'N Fit: Bronx nutrition and fitness initiative for teens
CHAM: Children's Hospital at Montefiore
StaRI: Standards for Reporting Implementation Studies
YD: Youth development
MSHP: Montefiore School Health Program
SBHC: School-Based Health Center
MMCC: Mosholu Montefiore Community Center
WITS: Wellness in the Schools
PIRC: Prevention Intervention Research Center
USDA: United States Department of Agriculture
PS/MS: Public school/middle school
MA: Medical Assistant
RN: Registered Nurse
CHO: Community Health Organizer
EMR: Electronic Medical Record
NHLBI: National Heart, Lung, and Blood Institute
NYC DYCD: New York City Department of Youth and Community Development
PI: Principal Investigator
HRSA: Heath Resources and Services Administration
HbA1C: Glycated hemoglobin test.

## References

[1] Ogden C. L., Carroll M. D., Lawman H. G. (2016). Trends in obesity prevalence among children and adolescents in the United States, 1988–1994 through 2013-2014. *JAMA*.

[2] Skinner A. C., Skelton J. A. (2014). Prevalence and trends in obesity and severe obesity among children in the United States, 1999–2012. *JAMA Pediatrics*.

[3] Seo D. C., King M. H., Kim N., Sovinski D., Meade R., Lederer A. M. (2015). Predictors for persistent overweight, deteriorated weight status, and improved weight status during 18 months in a school-based longitudinal cohort. *American Journal of Health Promotion*.

[4] Watson K. B., Harris C. D., Carlson S. A., Dorn J. M., Fulton J. E. (2016). Disparities in adolescents’ residence in neighborhoods supportive of physical activity—United States, 2011-2012. *Morbidity and Mortality Weekly Report*.

[5] Grossman D. C., Bibbins-Domingo K., Curry S. J. (2017). Screening for obesity in children and adolescents: US preventive services task force recommendation statement. *JAMA*.

[6] Seo D.-C., Sa J. (2010). A meta-analysis of obesity interventions among US minority children. *Journal of Adolescent Health*.

[7] Wilson D. K. (2009). New perspectives on health disparities and obesity interventions in youth. *Journal of Pediatric Psychology*.

[8] Whitlock E. P. (2005). Screening and interventions for childhood overweight: a summary of evidence for the US preventive services task force. *Pediatrics*.

[9] Dao H. H., Frelut M. L., Oberlin F., Peres G., Bourgeois P., Navarro J. (2004). Effects of a multidisciplinary weight loss intervention on body composition in obese adolescents. *International Journal of Obesity*.

[10] Chehab L. G., Pfeffer B., Vargas I., Chen S., Irigoyen M. (2007). “Energy up”: a novel approach to the weight management of inner-city teens. *Journal of Adolescent Health*.

[11] Evans R. K., Franco R., Stern M. (2009). Evaluation of a 6-month multi-disciplinary healthy weight management program targeting urban, overweight adolescents: effects on physical fitness, physical activity, and blood lipid profiles. *Pediatric Obesity*.

[12] Whitlock E. P., O’Connor E. A., Williams S. B., Beil T. L., Lutz K. W. (2010). Effectiveness of weight management interventions in children: a targeted systematic review for the USPSTF. *Pediatrics*.

[13] Laird Y., Fawkner S., Kelly P., McNamee L., Niven A. (2016). The role of social support on physical activity behaviour in adolescent girls: a systematic review and meta-analysis. *International Journal of Behavioral Nutrition and Physical Activity*.

[14] Woolford S. J., Sallinen B. J., Clark S. J., Freed G. L. (2011). Results from a clinical multidisciplinary weight management program. *Clinical Pediatrics*.

[15] Savoye M., Nowicka P., Shaw M. (2011). Long-term results of an obesity program in an ethnically diverse pediatric population. *Pediatrics*.

[16] DCCT (1996). Lifetime benefits and costs of intensive therapy as practiced in the diabetes control and complications trial. *JAMA*.

[17] Ickes M. J., McMullen J., Haider T., Sharma M. (2014). Global school-based childhood obesity interventions: a review. *International Journal of Environmental Research and Public Health*.

[18] Belansky E. S., Cutforth N., Delong E. (2009). Early impact of the federally mandated local wellness policy on physical activity in rural, low-income elementary schools in Colorado. *Journal of Public Health Policy*.

[19] Foster G. D., Sherman S., Borradaile K. E. (2008). A policy-based school intervention to prevent overweight and obesity. *Pediatrics*.

[20] Gortmaker S. L., Peterson K., Wiecha J. (1999). Reducing obesity via a school-based interdisciplinary intervention among youth: planet health. *Archives of Pediatrics and Adolescent Medicine*.

[21] Robinson T. N. (1999). Reducing children’s television viewing to prevent obesity: a randomized controlled trial. *JAMA*.

[22] Rieder J., Khan U. I., Heo M. (2013). Evaluation of a community-based weight management program for predominantly severely obese, difficult-to-reach, inner-city minority adolescents. *Childhood Obesity*.

[23] Peralta L. R., Jones R. A., Okely A. D. (2009). Promoting healthy lifestyles among adolescent boys: the fitness improvement and lifestyle awareness program RCT. *Preventive Medicine*.

[24] Weintraub D. L., Tirumalai E. C., Haydel K. F., Fujimoto M., Fulton J. E., Robinson T. N. (2008). Team sports for overweight children: the stanford sports to prevent obesity randomized trial (SPORT). *Archives of Pediatrics & Adolescent Medicine*.

[25] Dudley D. A., Okely A. D., Pearson P., Peat J. (2010). Engaging adolescent girls from linguistically diverse and low income backgrounds in school sport: a pilot randomised controlled trial. *Journal of Science and Medicine in Sport*.

[26] Eather N., Morgan P. J., Lubans D. R. (2016). Improving health-related fitness in adolescents: the CrossFit Teens™ randomised controlled trial. *Journal of Sports Sciences*.

[27] Christian D., Todd C., Hill R. (2016). Active children through incentive vouchers—evaluation (ACTIVE): a mixed-method feasibility study. *BMC Public Health*.

[28] Force C. P. S. T. (2017). *Obesity Prevention and Control: School-Based Programs*.

[29] Pinnock H., Barwick M., Carpenter C. R. (2017). Standards for reporting implementation studies (StaRI) statement. *BMJ*.

[30] Hester J. A., Stange P. V., Seeff L. C., Davis J. B., Craft C. A. (2015). *Toward Sustainable Improvements in Population Health: Overview of Community Integration Structures and Emerging Innovations in Financing*.

[31] Dietz W. H., Belay B., Bradley D. (2017). *A Model Framework that Integrates Community and Clinical Systems for the Prevention AND Management of Obesity and Other Chronic Diseases*.

[32] Roth J. L., Brooks-Gunn J. (2003). What exactly is a youth development program? Answers from research and practice. *Applied Developmental Science*.

[33] McKinney C., Bishop V., Cabrera K. (2014). NuFit: nutrition and fitness CBPR program evaluation. *Journal of Prevention and Intervention in the Community*.

[34] Stock S., Miranda C., Evans S. (2007). Healthy buddies: a novel, peer-led health promotion program for the prevention of obesity and eating disorders in children in elementary school. *Pediatrics*.

[35] Kanyamee M., Fongkaew W., Chotibang J., Aree P., Kennedy C. (2013). An intervention study of changing eating behaviors and reducing weight in thai children aged 10–12. *Pacific Rim International Journal of Nursing Research*.

[36] Katz D. L., O’Connell M., Njike V. Y., Yeh M. C., Nawaz H. (2008). Strategies for the prevention and control of obesity in the school setting: systematic review and meta-analysis. *International Journal of Obesity*.

[37] Jelalian E., Mehlenbeck R., Lloyd-Richardson E. E., Birmaher V., Wing R. R. (2006). Adventure therapy’ combined with cognitive-behavioral treatment for overweight adolescents. *International Journal of Obesity*.

[38] Eskicioglu P., Halas J., Sénéchal M. (2014). Peer mentoring for type 2 diabetes prevention in first nations children. *Pediatrics*.

[39] Story M. (1999). School-based approaches for preventing and treating obesity. *International Journal of Obesity and Related Metabolic Disorders*.

[40] Foundation R. W. J (2017). *Health Outcomes: Overall Rank*.

[41] Egger J. R., Bartley K. F., Benson L., Bellino D., Kerker B. (2009). Childhood obesity is a serious concern in New York City: higher levels of fitness associated with better academic performance. *NYC Vital Signs*.

[42] Public School 95 Sheila Mencher in Bronx, New York (2017). http://public-schools.startclass.com/l/61172/Public-School-95-Sheila-Mencher-in-Bronx-New-York.

[43] da Costa Nunez R., Adams M., Gleason A., Harris S. Failures and solutions: New Yorkers’ views on homelessness.

[44] Atkiss K., Moyer M., Desai M., Roland M. (2011). Positive youth development: an integration of the developmental assets theory and the socio-ecological model. *American Journal of Health Education*.

[45] *Education, N. Y. C. D. O. NYC Fitnessgram*.

[46] Barlow S. E. (2007). Expert committee recommendations regarding the prevention, assessment, and treatment of child and adolescent overweight and obesity: summary report. *Pediatrics*.

[47] (2017). *Service U. S. D. A. F. A. N. MyPlate*.

[48] Institute N. I. H. N. H. L. A. B. (2017). *How Much Sleep is Enough?*.

[49] Glasgow R. E., Vogt T. M., Boles S. M. (1999). Evaluating the public health impact of health promotion interventions: the RE-AIM framework. *American Journal of Public Health*.

[50] McGoey T., Root Z., Bruner M. W., Law B. (2016). Evaluation of physical activity interventions in children via the reach, efficacy/effectiveness, adoption, implementation, and maintenance (RE-AIM) framework: a systematic review of randomized and non-randomized trials. *Preventive Medicine*.

[51] Kessler R. S., Purcell E. P., Glasgow R. E., Klesges L. M., Benkeser R. M., Peek C. J. (2013). What does it mean to “employ” the RE-AIM model. *Evaluation & the Health Professions*.

[52] Gaglio B., Shoup J. A., Glasgow R. E. (2013). The RE-AIM framework: a systematic review of use over time. *American Journal of Public Health*.

[53] Mugavero M. J., Amico K. R., Horn T., Thompson M. A. (2013). The state of engagement in HIV care in the United States: from cascade to continuum to control. *Clinical Infectious Diseases*.

[54] O’Connor E. A., Evans C. V., Burda B. U., Walsh E. S., Eder M., Lozano P. (2017). Screening for obesity and intervention for weight management in children and adolescents: evidence report and systematic review for the US preventive services task force. *JAMA*.

[55] Epic (2017). http://www.Epic.com/software.

[56] Plan-Do-Study-Act (PDSA) Cycle (2017). https://innovations.ahrq.gov/qualitytools/plan-do-study-act-pdsa-cycle.

[57] Kaplan S. A., Calman N. S., Golub M., Davis J. H., Ruddock C., Billings J. (2006). Racial and ethnic disparities in health: a view from the South Bronx. *Journal of Health Care for the Poor and Underserved*.

[58] McKee M. D., O’Sullivan L. F., Weber C. M. (2006). Perspectives on confidential care for adolescent girls. *Annals of Family Medicine*.

[59] Education N. Y. C. D. O. STEM (2017). http://schools.nyc.gov/Academics/STEM/default.htm.

[60] Doran G. T. (1981). There’s a S.M.A.R.T. way to write management’s goals and objectives. *Management Review*.

[61] Cason-Wilkerson R., Goldberg S., Albright K., Allison M., Haemer M. (2015). Factors influencing healthy lifestyle changes: a qualitative look at low-income families engaged in treatment for overweight children. *Childhood Obesity*.

[62] Sallinen B. J., Schaffer S., Woolford S. J. (2013). In their own words: learning from families attending a multidisciplinary pediatric weight management program at the YMCA. *Childhood Obesity*.

[63] Puhl R. M., Peterson J. L., Luedicke J. (2013). Weight-based victimization: bullying experiences of weight loss treatment-seeking youth. *Pediatrics*.

